# Dr. Oscar J. Stafford

**Published:** 1916-06

**Authors:** 


**Dr. Oscar J. Stafford,** Buffalo 1858, died suddenly April 12, while attending a patient near his home in Port Chester, N. Y., aged 58. He was physician to the United Hospital of Port Chester.

